# Prevalence of severe-profound hearing loss in South Korea: a nationwide population-based study to analyse a 10-year trend (2006–2015)

**DOI:** 10.1038/s41598-018-28279-z

**Published:** 2018-07-02

**Authors:** Gi Jung Im, Joong Ho Ahn, Jun Ho Lee, Kyung do Han, Seung Hwan Lee, Jin-Sook Kim, Hyunsook Jang, Jong Woo Chung

**Affiliations:** 1 Department of Otolaryngology-Head and Neck Surgery, Korea University College of Medicine, Seoul, Korea; 2 Department of Otolaryngology-Head and Neck Surgery, Asan Medical Center, University of Ulsan College of Medicine, Seoul, Korea; 3 Department of Otolaryngology-Head and Neck Surgery, Seoul University College of Medicine, Seoul, Korea; 4 Department of Biostatistics, College of Medicine, Catholic University of Korea, Seoul, Korea; 5 Department of Otolaryngology-Head and Neck Surgery, Hanyang University College of Medicine, Seoul, Korea; 6 Division of Speech Pathology and Audiology, Hallym University College of Natural Sciences, Chuncheon, Korea

## Abstract

To estimate prevalence of severe-profound hearing loss (HL) in South Korea, and analyse a 10-year trend of HL according to age, sex, and region by using a nationwide population-based database. Retrospective review from Korean National Health Insurance Service from 2006 to 2015. The degree of severe-profound HL was classified into six grades, based mostly on HL worse than 60 dB HL for both ears. Absolute number of HL was the highest in 2011 (0.25 million; males, 0.14 million; females, 0.11 million); it decreased gradually until 2015. Total HL prevalence was the highest in 2010 (0.5%; 251,954), and decreased annually to 2015 (0.46%; 237,272). The trend of HL prevalence showed a gradual decrease from 2010 to 2015. Prevalence of severe-profound HL was always higher in the male population (1.19 times higher than female in 2015). Prevalence of HL was higher in rural areas than in urban areas (1.4 times higher in 2015). Number of severe-profound HL in South Korea decreased gradually in all age groups annually, even though some older age groups had the highest peak in 2010–2011. Prevalence of severe-profound HL decreases gradually in all age groups annually in South Korea, although the absolute number of HL cases increases rapidly among those aged over 80 years.

## Introduction

Hearing loss (HL) is an inability to hear sound or speech, and total HL is defined as deafness. The impact of HL is severe, resulting in (1) communication disorder, (2) exclusion from the social community and family, and (3) incurrence of costs associated with health insurance or national health service including hearing aids^1–4^. In the US, direct medical cost associated with HL ranged from $3.3 to 12.8 billion in 2015^5^. Thus, the prevention of HL is strongly recommended, because HL can be prevented in half of the cases, especially in children or in those caused by infections^6–8^.

The degree of HL has been classified using several methods, and the classifications made by the American Speech-Language-Hearing Association (ASHA) and World Health Organization (WHO) are widely used. According to the ASHA, slight HL is defined as HL ranging from 16 to 25 dB HL; mild HL, from 26 to 40 dB HL; moderate HL, from 41 to 55 dB HL; moderately severe HL, from 56 to 70 dB HL; severe HL, from 71 to 90 dB HL; and profound HL, >91 dB HL^9^. According to the WHO, “disabling HL” refers to HL > 40 dB HL in the better-hearing ear in adults and a HL > 30 dB HL in the better-hearing ear in children^10^. Normal hearing is defined as a threshold of 25 dB HL or better in both ears^10^. Mild HL is defined as HL ranging from 26 to 40 dB HL; moderate HL, from 41 to 60 dB HL; severe HL, from 61 to 80 dB HL, and profound HL, >81 dB HL^10^. Hard of hearing refers to individuals with HL ranging from mild to severe, who can benefit from hearing aids. Deaf individuals mostly have profound HL, and may benefit from cochlear implants^10^.

Recently, several nationwide studies on HL have been published from some nations such as South Korea, Japan, and Taiwan^11–13^. South Korea has some advantages when conducting a nationwide study on HL or on disease prevalence/incidence. The reason is that the territory is relatively small, transportation and accessibility to medical service is excellent, and the entire national population is registered with the National Health Insurance Service, with all medical data being well organised in their respective National Health Information Database (NHID). Thus, the analysis of nationwide data on HL can provide the true prevalence and trends of severe-profound HL in South Korea.

The aim of this study was to estimate the prevalence of severe-profound HL in South Korea, and to analyse a 10-year trend of HL according to age, sex, and region by using a nationwide population-based database. According to the degree of HL, nationwide registered data on severe-profound HL were classified into six grades, and the proportion of HL grades and a 10-year trend were analysed according to age groups.

## Methods

This study used nationwide data from the NHID, which is operated by the Korean National Health Insurance Service (KNHIS), a government-affiliated agency under the Korean Ministry of Health and Welfare that supervises all medical activities in Korea. All Korean citizens and registered foreigners are enrolled in and receive medical services from the KNHIS. Most patients with HL are registered in the national disability registry (NDR) of South Korea. This social registration is available for patients with severe-profound HL or deafness to provide them with disability benefits such as financial assistance for acquiring hearing aids or cochlear implants. In 2017, the Korean government provides financial support of approximately $1,000 for hearing aids to each registered person with disabling HL, and about 90% financial assistance for acquiring cochlear implants.

Retrospective medical data for patients of all ages were extracted from the NHID from January 2006 to December 2015 (NHIS-2016). Retrospective medical data from the NHID do not involve any personal data such as name, but just provide age, gender, number of patients, and the national classification code of disease. Thus, KNHIS approved the nationwide study without informed consent from each person.

The NHID and NDR contain information on patients’ demographics, degree of HL, medical service use, medication, deductions, and claims. According to the NDR, the degree of severe-profound HL is classified into six grades: 1st grade disabling HL (both-side HL ≥ 90 dB HL and speech disorder), 2nd grade (both-side HL ≥ 90 dB HL), 3rd grade (both-side HL ≥ 80 dB HL), 4th grade (both-side HL ≥ 70 dB HL), 5th grade (both-side HL ≥ 60 dB HL), and 6th grade (worse-side HL ≥ 80 dB HL and other-side HL ≥ 40 dB HL). Pure-tone thresholds were obtained using the pure-tone averages (PTAs) at four frequencies (0.5, 1, 2, and 4 kHz). To get registered in the NDR as having severe-profound HL, patients need to undergo PTA tests at least three times within an interval of 3–7 days, and the PTA test results must be confirmed using auditory brainstem response (ABR) or auditory steady-state response (ASSR). The KNHIS reviews the hearing test results and confirms registration to the NDR, and medical professors with expertise in otology review and debate over issues concerning the hearing test results. Thus, the analysis of the NDR can yield reliable information about the true prevalence of severe-profound HL in South Korea.

In this study, we analysed the sex, age, region, and yearly trends of the registered patients with disabling HL. To investigate the trend in HL over 10 years, we also examined the prevalence and incidence of disabling HL according to age, sex, and region. This study was conducted by the Research Committee of the Korean Society of Otorhinolaryngology-Head and Neck Surgery (www.korl.or.kr), and the Korean Audiological Society (www.audiosoc.or.kr) reviewed and confirmed the study. As a representative hospital, the institutional review board of the Korea University College of Medicine approved this study (KUMC IRB ED16185). All methods were performed in accordance with the relevant guidelines and regulations.

Statistical analysis was performed using SAS software version 9.3 (SAS Institute, Cary, NC). We analysed the age-standardized rates for the estimation of the exact prevalence and incidence. However, this nationwide study used actual data from the entire population of South Korea, and hence, no data on standard deviation were available, and there was little need to perform an analysis for predicting trends in a universal population. All data generated or analysed during this study are available from the corresponding author on reasonable request.

## Results

### The status of severe-profound HL in South Korea from 2006 to 2015

**Figure 1:**
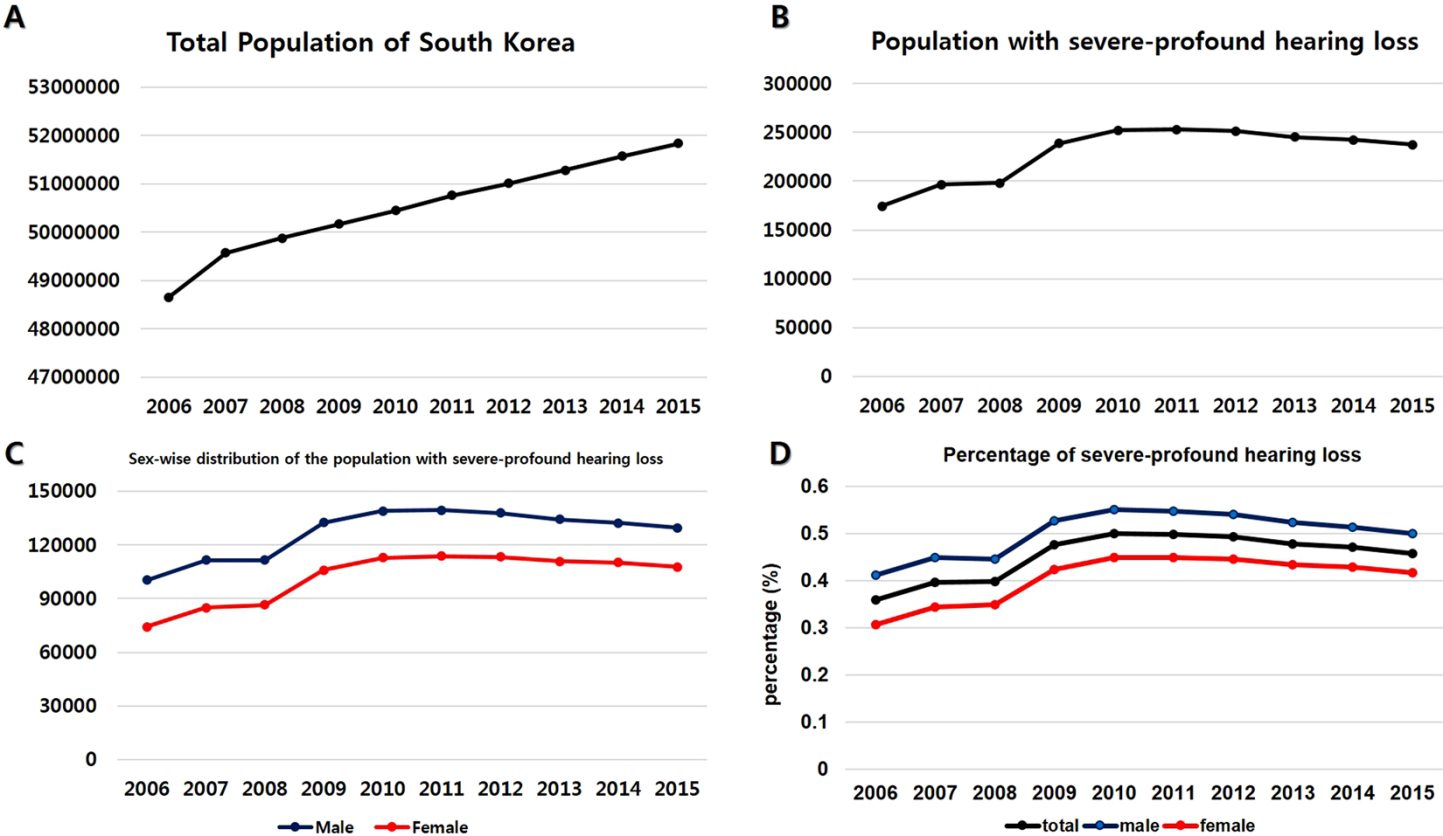
The status of severe-profound hearing loss (HL) in South Korea from 2006 to 2015. (**A**) The total population of South Korea continues to increase from 48.6 million to 51.8 million. (**B**) Population with severe-profound HL. (**C**) Sex-wise distribution of the population with severe-profound HL. Males have more severe-profound HL than do females. (**D**) The percentage of severe-profound HL using a nationwide population-based study to analyse a 10-year trend in hearing impairment. Total HL percentage of 2010 is 0.5%, which is the highest, and it decreases to 0.46% in 2015. The trend of HL percentage shows a gradual decrease from 2010 to 2015. The percentage of severe-profound HL is always higher in the male population than in the female population.

From 2006 to 2015, the total population of South Korea continued to increase from 48.6 million to 51.8 million. An approximate 0.7% annual increase was observed in the total population of South Korea (Fig. 1A). Korea’s per capita income also increased from $20,888 to $27,105 (from the data of international monetary fund). The population with severe-profound HL was 174,568 in 2006; the highest population was 253,008 in 2011; and the population gradually decreased to 237,272 in 2015 (Fig. 1B). Korea’s sex ratio is annually very similar at 1:1. However, the male population has more severe-profound HL than does the female population, and the trend analysis showed the highest peak in 2011 for both sexes, and the population with severe-profound HL gradually decreased until 2015 (Fig. 1C). Total percentage of HL was 0.5%; 251,954 (male: 0.55%; female: 0.45%) in 2010, which was the highest, and it subsequently decreased to 0.46%; 237,272 in 2015 (male: 0.5%; female: 0.42%). The trend of the percentage of HL showed a gradual decrease from 2010 to 2015. The prevalence of severe-profound HL was always higher in the male population than in the female population (Fig. 1D). Table 1 shows all raw data of total population, severe-profound HL with each grades, and the related percentages.

**Table 1:** The population and demographic data of total population and severe-profound hearing loss (HL) in South Korea from 2006 to 2015.

	2006	2007	2008	2009	2010	2011	2012	2013	2014	2015
Total Population	48656226	49568218	49879081	50167339	50444520	50761986	51011067	51280480	51573506	51834660
MALE	24419863	24862525	25013456	25149888	25281254	25433158	25537282	25664677	25811237	25933249
FEMALE	24236363	24705693	24865625	25017451	25163266	25328828	25473785	25615803	25762269	25901411
Total Hearing Loss	174568	196357	198074	238450	251954	253008	251186	245277	242472	237272
MALE	100350	111460	111478	132494	139058	139337	137914	134383	132255	129544
FEMALE	74217	84896	86596	105956	112896	113671	113272	110880	110168	107728
Grade 1	3342	4673	4972	5853	6366	6431	6542	6511	6518	7208
Grade 2	42766	44542	43886	46734	46313	45682	45152	44168	43518	42949
Grade 3	31230	35756	36133	44775	44791	44198	43432	42164	40999	39740
Grade 4	34146	38862	39395	49933	55486	56235	55627	53977	53338	51663
Grade 5	32466	39482	40603	54059	60162	61304	61485	60145	59937	58431
Grade 6	30618	33042	33085	37096	38836	39158	38948	38312	38162	37281
Percent, total	0.359%	0.396%	0.397%	0.475%	0.499%	0.498%	0.492%	0.478%	0.470%	0.458%
Percent, male	0.411%	0.448%	0.446%	0.527%	0.550%	0.548%	0.540%	0.524%	0.512%	0.500%
Percent, female	0.306%	0.344%	0.348%	0.424%	0.449%	0.449%	0.445%	0.433%	0.428%	0.416%

^1^Both-side HL ≥ 90 dB HL and speech disorder, ^2^both-side HL ≥ 90 dB HL, ^3^both-side HL ≥ 80 dB HL, ^4^both-side HL ≥ 70 dB HL, ^5^both-side HL ≥ 60 dB HL, ^6^worse-side HL ≥ 80 dB HL and other-side HL ≥ 40 dB HL. Total severe-profound HL is the sum of grade 1–6.

### The degree of severe-profound HL

**Figure 2:**
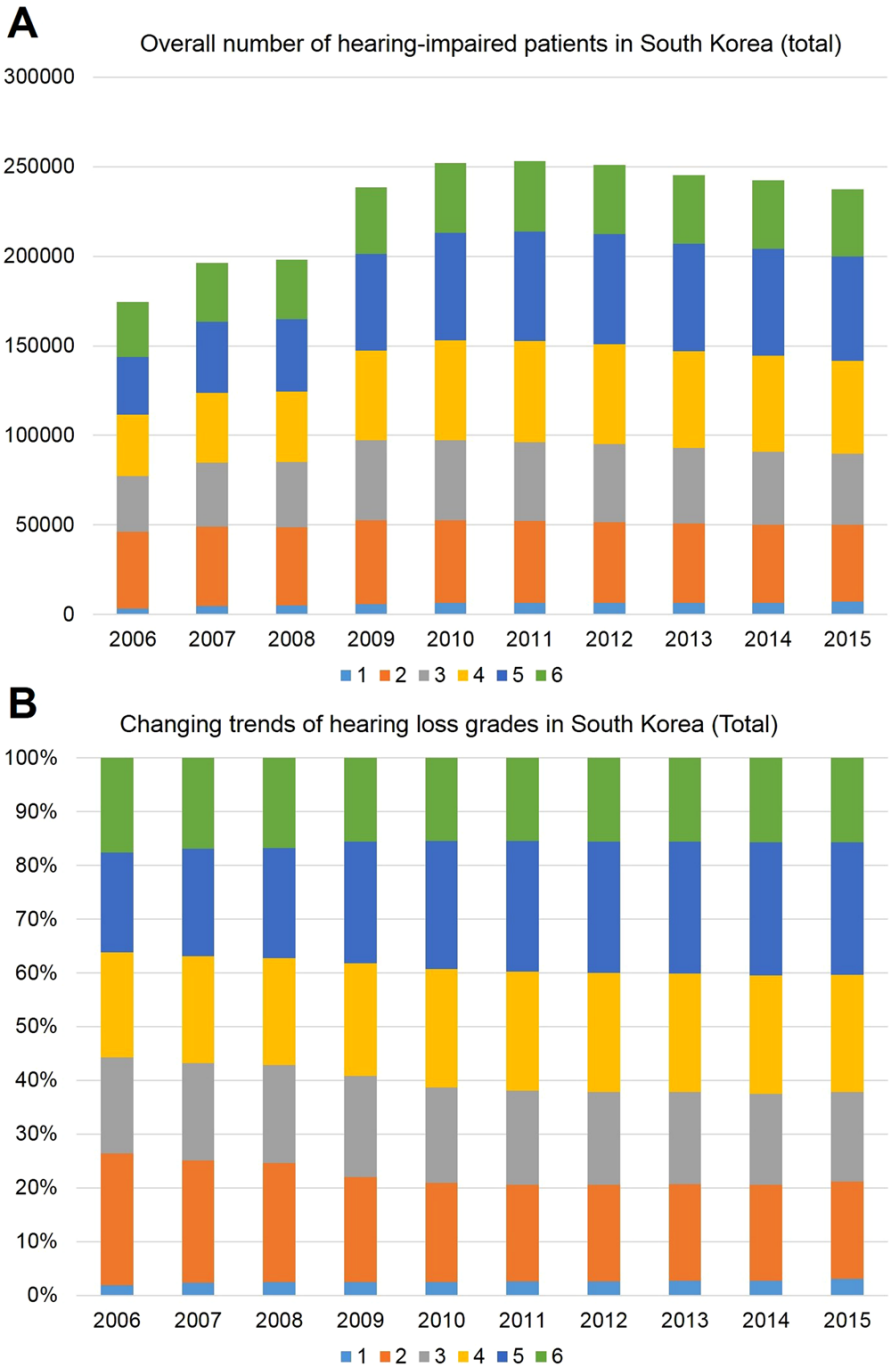
The total number of patients with severe-profound hearing loss (HL) in South Korea determined using a nationwide population-based study to analyse a 10-year trend in hearing impairment. (**A**) The number is the highest in 2011 (0.25 million individuals), and decreases gradually until 2015. (**B**) From 2006 to 2015, the prevalence of 1st grade HL increased from 1.91% to 3.04%; 2nd grade HL decreased from 24.5% to 18.1%; 3rd grade HL decreased from 17.89% to 16.75%; 4th grade HL increased from 19.56% to 21.77%; 5th grade HL increased from 18.6% to 24.63%; and 6th grade HL decreased from 17.54% to 15.71%. ^1^both-side HL ≥ 90 dB HL and speech disorder, ^2^both-side HL ≥ 90 dB HL, ^3^both-side HL ≥ 80 dB HL, ^4^both-side HL ≥ 70 dB HL, ^5^both-side HL ≥ 60 dB HL, ^6^worse-side HL ≥ 80 dB HL and other-side HL ≥ 40 dB HL.

The prevalence of severe-profound HL was the highest in 2011 (0.25 million individuals), and decreased gradually until 2015 (Fig. 2A). In the NDR, six grades were used to classify patients with HL. From 2006 to 2015, the prevalence of 1st grade HL increased from 1.91% to 3.04%; 2nd grade HL decreased from 24.5% to 18.1%; 3rd grade HL decreased from 17.89% to 16.75%; 4th grade HL increased from 19.56% to 21.77%; 5th grade HL increased from 18.6% to 24.63%; and 6th grade HL decreased from 17.54% to 15.71% (Fig. 2B).

### The prevalence of severe-profound HL in the male population

**Figure 3:**
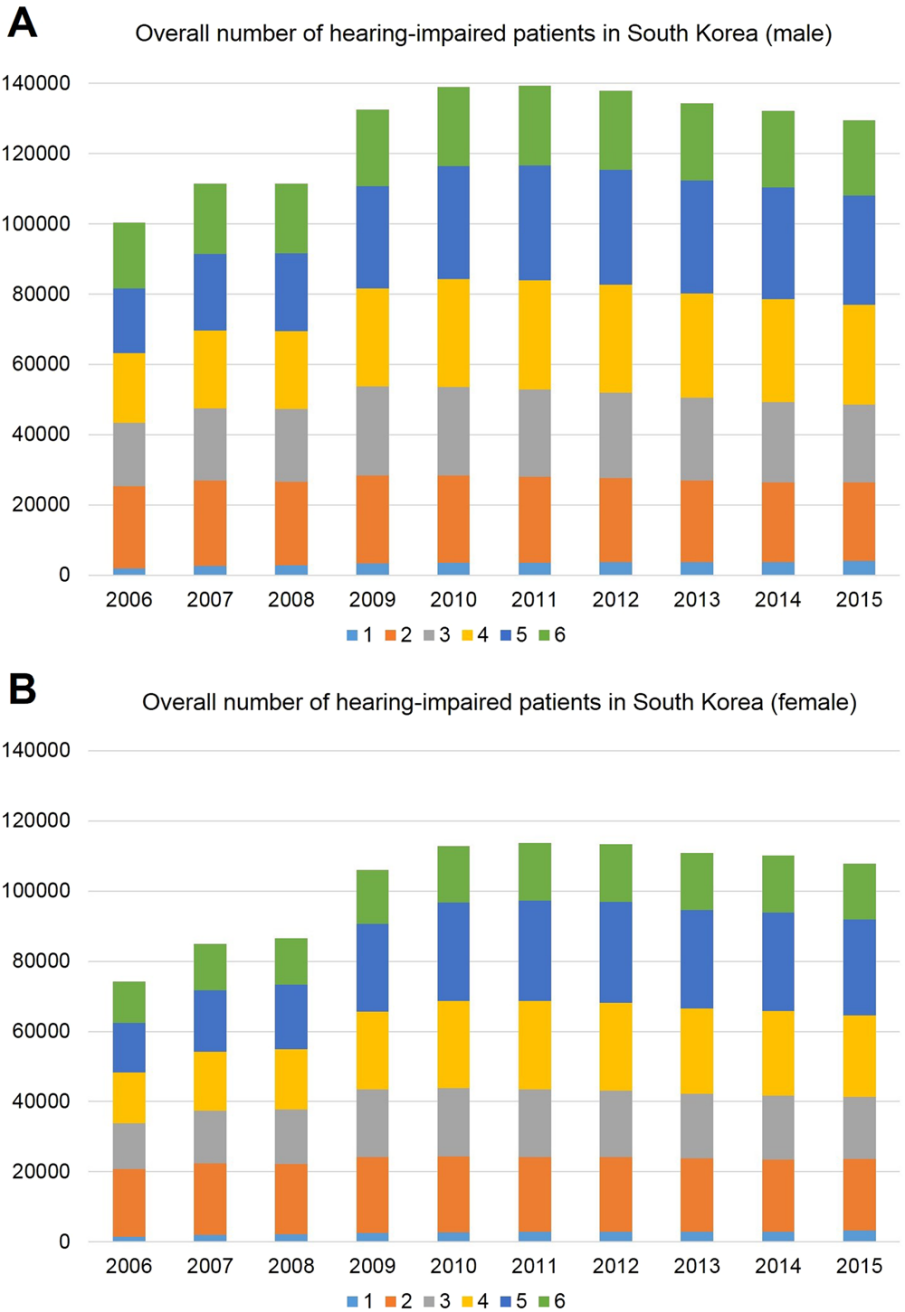
Number of patients with severe-profound hearing loss (HL) in the male and female population determined using a nationwide population-based study to analyse a 10-year trend in hearing impairment. (**A**) The prevalence in the male is the highest in 2011 (0.14 million individuals), and decreases gradually until 2015. (**B**) The prevalence in the female is the highest in 2011 (0.11 million individuals), and decreases gradually until 2015. ^1^both-side HL ≥ 90 dB HL and speech disorder, ^2^both-side HL ≥ 90 dB HL, ^3^both-side HL ≥ 80 dB HL, ^4^both-side HL ≥ 70 dB HL, ^5^both-side HL ≥ 60 dB HL, ^6^worse-side HL ≥ 80 dB HL and other-side HL ≥ 40 dB HL.

The prevalence of severe-profound HL in the male population was 100,350 (0.411%) in 2006, with the highest peak of 139,337 (0.548%) in 2011, and decreasing gradually to 129,544 (0.5%) in 2015 (Fig. 3A). According to the degree of disabling HL, the pattern of change in NDR grades in the male population was similar to that in the total population: grades 1, 4, and 5 showed an annual increase, whereas grades 2, 3, and 6 showed an annual decrease (Fig. 3A).

### The prevalence of severe-profound HL in the female population

The prevalence of severe-profound HL in the female population was 74,217 (0.306%) in 2006, with the highest peak of 113,671 (0.449%) in 2011, and decreasing gradually to 107,728 (0.416%) in 2015 (Fig. 3B). According to the degree of disabling HL, the pattern of change in NDR grades in the female population was similar to that in the total population: grades 1, 4, and 5 showed an annual increase, whereas grades 2, 3, and 6 showed an annual decrease (Fig. 3B).

### The regional distribution of age-standardized prevalence of severe-profound HL in South Korea: higher HL prevalence was shown in rural areas than in urban areas

**Figure 4:**
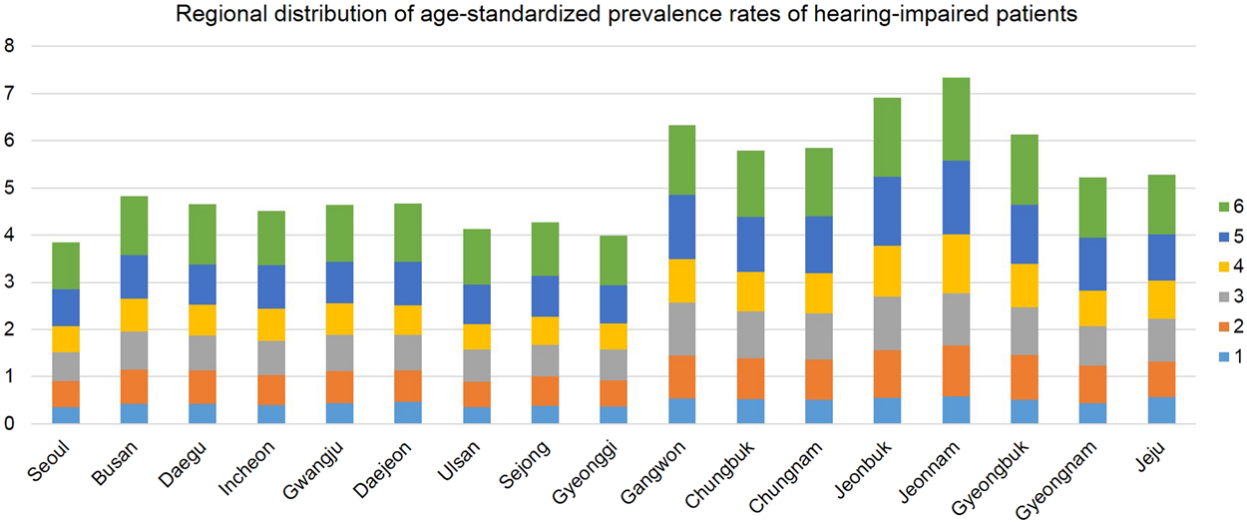
The regional distribution of age-standardized prevalence of severe-profound hearing loss (HL) in South Korea. Higher HL prevalence is shown in rural areas (eight latter areas) than in urban areas (nine initial cities or towns). Age-standardized prevalence of severe-profound HL is the highest in Jeonnam province, followed by Joenbuk province. The lowest prevalence is in Seoul, the capital of South Korea. Average of age-standardized prevalence value is 6.11 in rural areas and 4.39 in urban areas. ^1^both-side HL ≥ 90 dB HL and speech disorder, ^2^both-side HL ≥ 90 dB HL, ^3^both-side HL ≥ 80 dB HL, ^4^both-side HL ≥ 70 dB HL, ^5^both-side HL ≥ 60 dB HL, ^6^worse-side HL ≥ 80 dB HL and other-side HL ≥ 40 dB HL. For more details, see the supplementary file that is uploaded online.

Using the representative data from the year 2015, we evaluated the regional distribution of HL prevalence. Figure 4 shows a higher HL prevalence in rural areas (eight latter areas) than in urban areas (nine initial cities or towns). Age-standardized prevalence of severe-profound HL was the highest in Jeonnam province (7.34), followed by Joenbuk province (6.91). The lowest prevalence was shown in Seoul (3.84), the capital of South Korea. The average age-standardized prevalence was 6.11 in rural areas and 4.39 in urban areas (Fig. 4).

### Number of patients with severe-profound HL according to age groups

**Figure 5:**
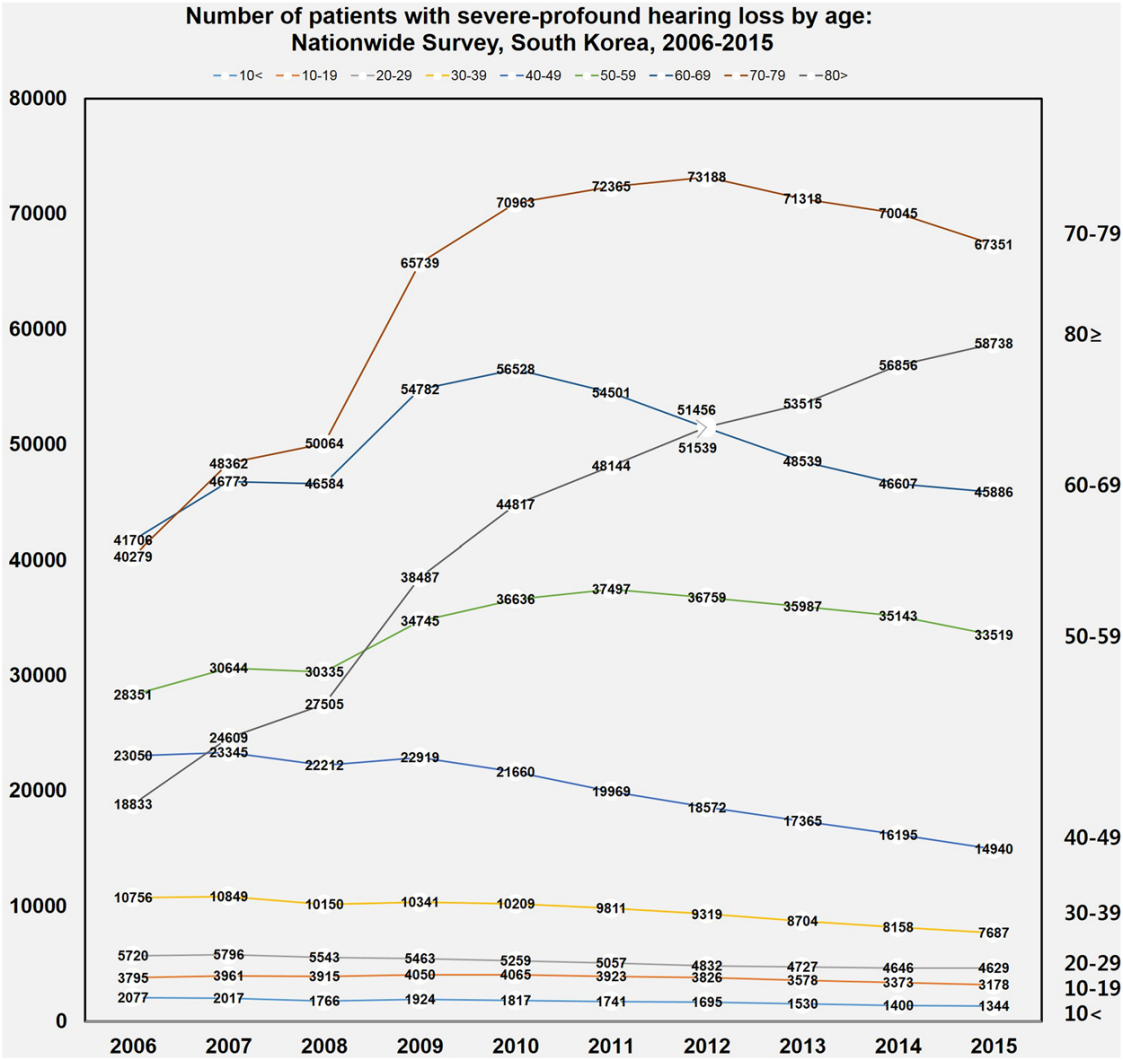
Number of individuals with severe-profound hearing loss (HL) in South Korea determined using a nationwide population-based study to analyse a 10-year trend according to age groups. In HL patients younger than 50 years, the number of HL cases decrease annually in all age groups (<10, 10 s, 20 s, 30 s, and 40 s). Among those in the 50 s, the number of HL cases is the highest at 37,497 in 2011, decreasing annually thereafter. Among those in the 60 s, the number of HL cases is the highest at 56,528 in 2010, decreasing annually thereafter. Among those in the 70 s, the number of HL cases is the highest at 73,188 in 2012, decreasing annually thereafter. However, the number of HL cases increases rapidly among those aged over 80 years (18,833 in 2006 to 58,738 in 2015).

According to age groups, the prevalence of severe-profound HL in South Korea was analysed using the NDR to evaluate a 10-year trend (Fig. 5). In HL patients younger than 50 years, the prevalence of HL decreases annually in all age groups (<10, 10 s, 20 s, 30 s, and 40 s). Among those in the 50 s, the prevalence of HL was the highest at 37,497 in 2011, decreasing annually thereafter. Among those in the 60 s, the prevalence of HL was the highest at 56,528 in 2010, decreasing annually thereafter. Among those in the 70 s, the prevalence of HL was the highest at 73,188 in 2012, decreasing annually thereafter. However, among those aged over 80 years, the prevalence of HL increased rapidly (18,833 in 2006 to 58,738 in 2015).

### The prevalence of severe-profound HL according to age groups

**Figure 6:**
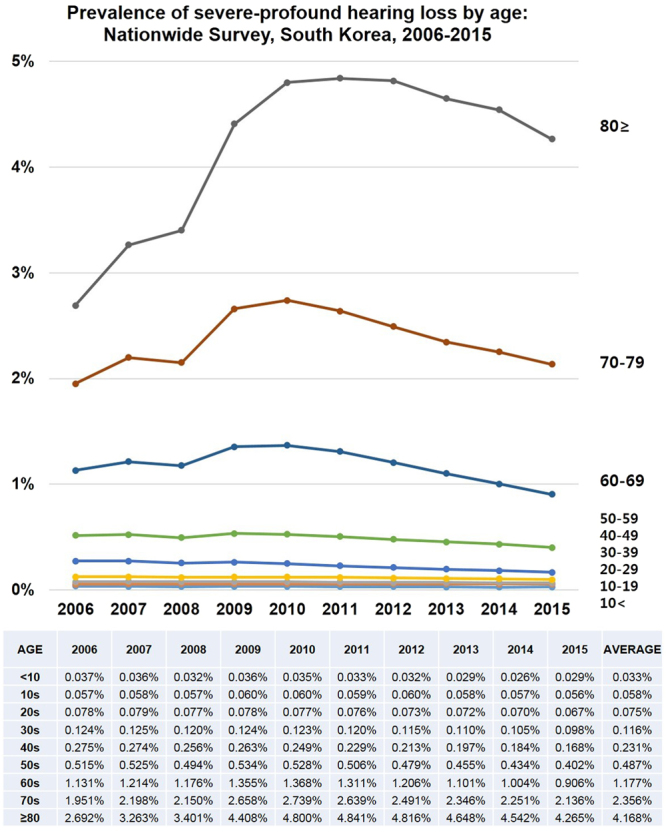
Prevalence of severe-profound hearing loss (HL) in South Korea determined using a nationwide population-based study to analyse a 10-year trend according to age groups. From the youngest group to the oldest group, the prevalence of severe-profound HL increases exponentially from 0.033% to 4.168%. In HL patients younger than 60 years, the prevalence of HL decreases annually in all age groups (<10, 10 s, 20 s, 30 s, 40 s, and 50 s). Among those in the 60 s, the prevalence of HL is the highest at 1.368% in 2010, decreasing annually thereafter. Among those in the 70 s, the prevalence of HL is the highest at 2.739% in 2010, decreasing annually thereafter. Among those in the 80 s, the prevalence of HL is the highest at 4.841% in 2011, decreasing annually thereafter, although the absolute number of HL cases increases rapidly among those aged over 80 years. Thus, the prevalence of severe-profound HL decreases gradually in all age groups annually in South Korea.

Figure 6 shows the prevalence of severe-profound HL in South Korea by age. From the youngest group to the oldest group, the prevalence of severe-profound HL exponentially increased from 0.033% to 4.168%. Of course, older people had higher prevalence of HL than younger people did. In HL patients younger than 60 years, the prevalence of HL decreased annually in the age groups (<10, 10 s, 20 s, 30 s, 40 s, and 50 s). Among those in the 60 s, the prevalence of HL was the highest at 1.368% in 2010, decreasing annually thereafter. Among those in the 70 s, the prevalence of HL was the highest at 2.739% in 2010, decreasing annually thereafter. Among those in the over 80 s, the prevalence of HL was the highest at 4.841% in 2011, decreasing annually thereafter. However, the absolute number of patients with HL increased rapidly in the group aged over 80 years. Thus, the prevalence of severe-profound HL decreased gradually in all age groups annually in South Korea, although some older age groups (60 s, 70 s, and ≥80) had the highest peak in 2010–2011.

## Discussion

This study is the first official big data analysis on HL in South Korea using a nationwide database, the NHID operated by the KNHIS, and conducted by the Korean Audiological Society. South Korea is an ideal country for performing nationwide big data analysis, because of its small territory, excellent transportation, easy accessibility to medical service, and electronic registration of medical activities of all nationals to the KNHIS. This study surveyed the entire population of over 50 million in South Korea, and not just a representative population or a part of the national population. This study shows clearly the “turning point” of HL prevalence in Korea.

South Korea has advanced from being a developing country and has joined the ranks of advanced countries. The economy of South Korea has been growing ($1.761 trillion in 2015; ranked 11th in the world), and the annual per capita income has been increasing from $20,888 in 2006 to $27,105 in 2015. Accordingly, medical services and disease prevention have also been improving; however, this has led to a rapidly “aging society” in South Korea. Therefore, it is important and valuable to analyse the trend of severe-profound HL according to age, sex, region, and the degree of disabling HL in South Korea.

In this study, the population with severe-profound HL hit the peak at some time point (2010–2011 in South Korea) and decreased gradually thereafter, even though the total population of South Korea as well as the aged population continued to increase during 2006–2015. The total percentage of severe-profound HL in 2010 hit the highest peak 0.5% (male: 0.55%; female: 0.45%), and it decreased to 0.46% (male: 0.5%; female: 0.42%) in 2015. According to the WHO global burden of HL in the year 2000, the prevalence of severe-profound HL (HL > 60 dB) was 0.4–4.5%^14^. In 2012, the WHO released data about disabling HL (HL > 40 dB in the better-hearing ear in adults and HL > 30 dB in the better-hearing ear in children), showing there are 360 million patients with disabling HL, which is 5.3% of the world’s population^15^. In the study of global and regional hearing impairment prevalence, hearing impairment was positively related to age, male sex, and middle- and low-income regions^15,16^. The global prevalence of hearing impairment (HL > 35 dB HL in the better ear) in 2008 was 1.4% for children aged 5–14 years, 9.8% for females >15 years of age and 12.2% for males >15 years of age^16^.

In advanced economies or high-income regions, HL among patients aged >65 years was 0.6%, which was comparable to data from South Korea (0.46–0.5%), and HL among patients aged >65 years was 1.2% globally^16^. According to the 2012 WHO data, the prevalence of HL in children decreases exponentially as the annual per capita income increases^15^. In adults aged ≥65 years, the prevalence of HL decreases exponentially as income increases^15^. Within the US, individuals with HL had 1.58 times higher odds of low income and 3.21 times higher odds of low educational attainment^17^. Thus, it seems that the prevalence of severe-profound HL in South Korea also decreases as the income increases.

In this study, the male population definitely has more severe-profound HL than did the female population, and the male/female prevalence ratio reached its peak at 1.22 in 2011 and at 1.19 in 2015. The WHO estimates also described a male/female prevalence ratio of 1.0–1.5 in 20 countries^14^. Another study suggested that the hearing threshold elevation in males appeared more rapid than in females, especially among those in the 30 s, 40 s, and 50 s^18^. According to data from the National Health and Nutrition Examination Survey (1999–2004) on US adults, the male/female prevalence ratio was 1.15 (53.4/46.6; HL > 25 dB), and bilateral HL in males appeared more substantial than that in females, especially among those in the 50 s and 60 s^19^. In a large cohort study in the Netherlands, the male/female prevalence ratio was 1.14 (33/29; HL > 35 dB) among participants aged ≥65 years^20^. the male/female prevalence ratio are expected to be smaller over time, as the nation develop; economy grows; income increases; aging society grows.

In the total population with severe-profound HL, an annual increase was observed in NDR grades 1, 4, and 5, whereas an annual decrease was observed in NDR grades 2, 3, and 6. The precise reasons behind this finding are unknown. However, we can speculate the reason that an increased interest in vocal and speech production may have increased the proportion of patients with 1st grade disabling HL (both-side HL ≥ 90 dB and speech disorder), and that an increased desire for hearing aids may have increased the proportion of patients with 4th grade (both-side HL ≥ 70 dB) and 5th grade (both-side HL ≥ 60 dB) HL. The decrease in the number of patients with NDR grades 2, 3, and 6 probably indicated a trend of decline in severe HL and asymmetric HL caused by inflammatory otologic disorders.

The average age-standardized prevalence value was 6.11 in rural areas and 4.39 in urban areas. Thus, the prevalence of severe-profound HL in rural areas was about 1.4 times higher than that in urban areas. Interestingly, higher HL prevalence was observed in rural areas than in urban areas, although cities have more noise sources than do rural areas. We speculate that patients in urban areas have greater accessibility to medical services, and this is enough to prevent HL or other otologic problems. Barriers in access to health care significantly affect the health outcomes of rural patients, and average driving distance to the health service among rural respondents was 4 times that of urban respondents^21,22^. Possible reason for this finding may be due to faster aging society in rural areas, but this study data was the average age-standardized prevalence. Especially in children and adolescents, there have been concerns about increasing levels of hearing impairment, in relation to noise exposure using personal devices regardless of regional variation, because even mild levels of hearing loss can affect educational outcomes^23^.

The prevalence of severe-profound HL increased exponentially from 0.033% (patients aged < 10 years) to 4.168% (patients aged ≥ 80 years) according to aging (Fig. 6)^24,25^. The absolute number of patients with HL annually decreased in all age groups, especially in the age groups <50 years of age. However, the number of patients with HL increased rapidly in the group aged over 80 years, which is a definite evidence of an “population ageing” in South Korea from 2006 to 2015. In 2015, people aged 65 or older now make up 13.1% of the population in South Korea, and the ratio will reach 24.3% in 2030. The prevalence of severe-profound HL decreased gradually in all age groups annually in South Korea, even though South Korea had a rapidly aging population. As the nation advanced and the incomes increased, the prevalence of severe-profound HL definitely decreased in all age groups, but the older age groups still had the highest peaks at some time points (2010–2011)^17,26–30^.

In conclusion, the total prevalence of severe-profound HL was 0.46% (237,272 in 2015) in South Korea. The prevalence of HL in 2015 was higher in the male population (male: 0.5%; female: 0.42%; 1.19 times higher than that in the female population) and in rural areas (1.4 times higher than that in urban areas). The number of patients with HL decreased annually in all age groups, especially in the age groups <50 years of age, from 2006 to 2015. However, the number of patients with HL increased rapidly in those aged over 80 years, because of population ageing. The prevalence of severe-profound HL in South Korea decreased gradually in all age groups annually, and some older age groups passed the highest peak of prevalence in 2010–2011.

### Limitations

In this study, a possible limitation is that some individuals may not be registered to the NDR of HL, and the prevalence of severe-profound HL may be underestimated. However, the KNHIS covers the entire national population in principle; hence, the trend of changes in HL prevalence observed in this study is solid. The other limitation is that the nationwide prevalence of mild-moderate HL cannot be estimated, because hearing tests and support for hearing aid still focus on registered HL groups (severe-profound HL). In the future, welfare paradigm should be tailored to different life stages to offer necessary hearing tests, HL prevention, and support for assistances.

### Further recommendations for research

In each age groups, changing trends of HL grades can be evaluated in the future. Further research might focus on the relations with otologic surgeries in the patients of severe-profound HL. In addition, the relationship between HL and associated disease may be analysed using comparison between whole HL group and age-sex matched 5-fold population without HL as control group.

## Electronic supplementary material

Dataset 1
