# Supplementary material for: Prevalence of severe-profound hearing loss in South Korea: a nationwide population-based study to analyse a 10-year trend (2006–2015)
Source: Sci Rep. 2018 Jul 2;8:9940. doi: 10.1038/s41598-018-28279-z (PMC6028585; doi:10.1038/s41598-018-28279-z)
Supplement: Supplementary file 1 — Dataset 1 [file 41598_2018_28279_MOESM1_ESM.docx]

**Prevalence of severe-profound hearing loss in South Korea: a nationwide population-based study to analyse a 10-year trend (2006**–**2015)**

**Gi Jung Im^1^, Joong Ho Ahn^2^, Jun Ho Lee^3^, Kyung do Han^4^, Seung Hwan Lee^5^, Jin-Sook Kim^6^, Hyunsook Jang^6^, Jong Woo Chung^2,^***

^1^Department of Otolaryngology-Head and Neck Surgery, Korea University College of Medicine, Seoul, Korea

^2^Department of Otolaryngology-Head and Neck Surgery, Asan Medical Center, University of Ulsan College of Medicine, Seoul, Korea

^3^Department of Otolaryngology-Head and Neck Surgery, Seoul University College of Medicine, Seoul, Korea

^4^Department of Biostatistics, College of Medicine, Catholic University of Korea, Seoul, Korea

^5^Department of Otolaryngology-Head and Neck Surgery, Hanyang University College of Medicine, Seoul, Korea

^6^Division of Speech Pathology and Audiology, Hallym University College of Natural Sciences, Chuncheon, Korea

*Corresponding author:

Jong Woo Chung, MD, PhD

Professor

^2^Department of Otolaryngology-Head and Neck Surgery, Asan Medical Center, University of Ulsan College of Medicine, Seoul, Korea

Tel: 82(Korea)-2-920-5486

Fax: 82(Korea)-2-925-5233

E-mail: gfinderjw@gmail.com, earkorea@gmail.com

**Gi Jung Im, logopas@korea.ac.kr, earkorea@gmail.com**

**Joong Ho Ahn, ucanhear@gmail.com**

**Jun Ho Lee, junlee@snu.ac.kr**

**Kyung do Han, hkd917@naver.com**

**Seung Hwan Lee, shlee711@gmail.com**

**Jin-Sook Kim, jskim@hallym.ac.kr**

**Hyunsook Jang, hsjang@hallym.ac.kr**

**Jong Woo Chung, gfinderjw@gmail.com**

Additional table related to Figure 4, the regional distribution of age-standardized prevalence of severe-profound hearing loss (HL) in South Korea. The table shows a higher HL prevalence in rural areas (eight latter areas) than in urban areas (nine initial cities or towns).

|  | **Grade 1** | **Grade 2** | **Grade 3** | **Grade 4** | **Grade 5** | **Grade 6** | **Total** |
| --- | --- | --- | --- | --- | --- | --- | --- |
| **Seoul** | 0.353 | 0.554 | 0.608 | 0.560 | 0.774 | 0.989 | **3.838** |
| **Busan** | 0.425 | 0.718 | 0.809 | 0.699 | 0.924 | 1.244 | **4.819** |
| **Daegu** | 0.424 | 0.711 | 0.741 | 0.647 | 0.859 | 1.271 | **4.652** |
| **Incheon** | 0.398 | 0.629 | 0.736 | 0.672 | 0.921 | 1.162 | **4.519** |
| **Gwangju** | 0.443 | 0.679 | 0.759 | 0.665 | 0.882 | 1.215 | **4.643** |
| **Daejeon** | 0.459 | 0.680 | 0.746 | 0.620 | 0.925 | 1.235 | **4.665** |
| **Ulsan** | 0.354 | 0.540 | 0.681 | 0.534 | 0.847 | 1.167 | **4.124** |
| **Sejong** | 0.377 | 0.621 | 0.679 | 0.587 | 0.868 | 1.139 | **4.271** |
| **Gyeonggi** | 0.359 | 0.563 | 0.647 | 0.560 | 0.806 | 1.056 | **3.991** |
| **Gangwon** | 0.543 | 0.899 | 1.124 | 0.926 | 1.363 | 1.472 | **6.327** |
| **Chungbuk** | 0.519 | 0.869 | 0.991 | 0.838 | 1.167 | 1.402 | **5.785** |
| **Chungnam** | 0.515 | 0.852 | 0.969 | 0.856 | 1.207 | 1.456 | **5.854** |
| **Jeonbuk** | 0.553 | 1.003 | 1.135 | 1.083 | 1.465 | 1.671 | **6.910** |
| **Jeonnam** | 0.577 | 1.075 | 1.117 | 1.245 | 1.562 | 1.768 | **7.343** |
| **Gyeongbuk** | 0.514 | 0.945 | 1.004 | 0.922 | 1.254 | 1.497 | **6.136** |
| **Gyeongnam** | 0.442 | 0.786 | 0.838 | 0.757 | 1.115 | 1.287 | **5.225** |
| **Jeju** | 0.566 | 0.745 | 0.918 | 0.799 | 0.986 | 1.271 | **5.285** |
